# Congenital Agenesis of the Olfactory Bulbs: What to Suspect?

**DOI:** 10.7759/cureus.12659

**Published:** 2021-01-12

**Authors:** Isabel Costa, Berta Rodrigues, Luís Dias

**Affiliations:** 1 Otorhinolaryngology and Head and Neck Surgery Department, Hospital de Braga, Braga, PRT

**Keywords:** olfactory bulb, isolated congenital anosmia, olfaction disorders, anosmia

## Abstract

Complete agenesis of the olfactory bulbs (OB) constitutes a rare cause of congenital anosmia, which is more often associated with cerebral malformations or genetic disorders.

The authors present a very rare case of a 28-year-old caucasian male with complaints of complete lack of the sense of smell since childhood. Radiologic study confirmed complete bilateral agenesis of the OB, with no other radiologic or clinical findings, such as delayed sexual development. Laboratorial investigation confirmed no signs of adrenocortical insufficiency.

Despite being immature in the term neonate, the OB are already functional at birth. Congenital agenesis of the OB can occur as an isolated deformity. On the other hand, it is a constant feature of several genetic syndromes, such as Kallmann’s syndrome or alobar holoprosencephaly. Most cases of isolated congenital anosmia occur sporadically, with no family history of the condition. Given the fact that no curative treatment is available, counselling for daily living precautions should be given to all patients. This is one of the first case reports about complete and isolated agenesia of the OB diagnosed in adulthood. The authors highlight the need to exclude several genetic syndromes that may be associated, albeit in a dissimulated way.

## Introduction

Complete agenesis of the olfactory bulbs (OB), also known as arrhinencephaly, constitutes a quite rare cause of congenital anosmia. Its incidence is estimated at 700 per 100,000 births. When present, anosmia is the unique nasal complaint and it is always non-progressive. This condition may occur as an isolated defect (not associated with additional symptoms) or it can be associated with other rare cerebral malformations or genetic disorders. Kallmann’s syndrome, with a prevalence of 1 in 10,000 men, is the most likely differential diagnosis but it is associated with isolated gonadotropin-releasing hormone deficiency and delayed sexual development [1,2].

Isolated congenital anosmia is a very rare condition characterized by a complete smelling defect that is present from birth in otherwise normal subjects. Complete agenesis of the OB in adults without any other clinical and radiological findings and with normal sexual characteristics is very difficult to be detected. However, clinical evidence of absent olfaction and MRI are sufficient for diagnosis of OB agenesis or dysgenesis. Molecular studies can also reveal specific genetic mutations [3-5]. In such cases, acquired causes of anosmia, such as fracture of the cribriform plate, viral-induced anosmia, intracranial tumors, olfactory groove meningiomas, vascular anomalies (i.e. a persistent fetal olfactory artery), and post-chemoradiation anosmia should also be considered and excluded, especially in these group age [5-6].

So far, and to the best of our knowledge, there are only a few published case reports about isolated congenital anosmia associated with complete agenesia of the OB detected in adulthood [1]. Here we present a very rare case of a complete congenital and isolated agenesis of the OB of a 28-year-old male. By reviewing the available literature, the authors aim to reflect on the best way to approach these patients, as well as on differential diagnoses that we should search for when faced with the suspicion of a congenital form of anosmia.

## Case presentation

A 28-year-old Caucasian man, an informatics engineer, with no relevant personal or family medical history, was refereed from his family doctor to Otorhinolaryngology evaluation for complaints of complete lack of the sense of smell since childhood. The patient and his family noticed the problem since childhood, but the symptom was always devalued. There was no previous history of trauma, drugs consumption or smoking habits, exposure to dust, woods, paint mists or other chemical agents or history of a previous nasal surgery. The patient had normal development and sexual function and activity. He had no history of trauma, surgery, chemical exposure or infection.other nasal complaints, namely nasal obstruction, rhinorrhea, frontal headache or epistaxis. He denied positive family history for any genetic disorder.

Otorhinolaryngologic examination was normal. There were no endoscopic nasal signs of trauma, rhinosinusitis, septal deviation or other anatomic malformations, nasal masses, rhinorrhea or foreign bodies. Sexual and cognitive development were also normal and neurologic exam found no evidence of central or other cranial nerves impairment.

**Figure 1:**
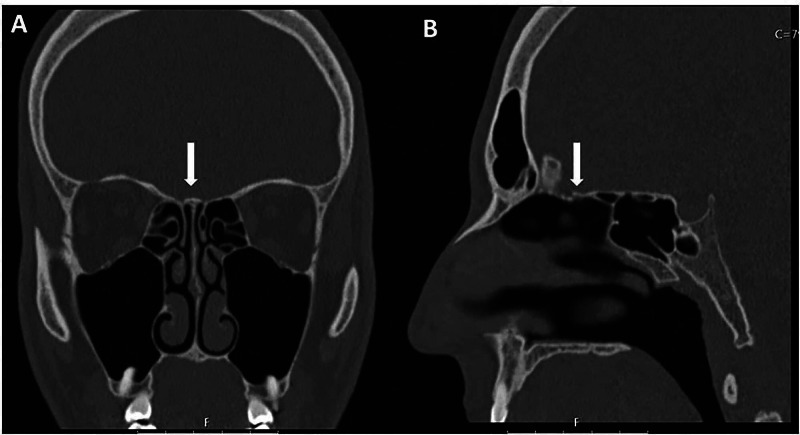
Cerebral and Paranasal Sinuses CT scan The CT scan did not reveal any anatomical anomalies of the cranium or facial massif. The nose and peri-nasal sinuses showed normal configuration and transparency, with no evidence of obstructive lesions. The region corresponding to the olfactory bulbs (arrow) did not show changes in density suggestive of expansive lesions or fracture lines (A - coronal cut; B - axial cut)

**Figure 2:**
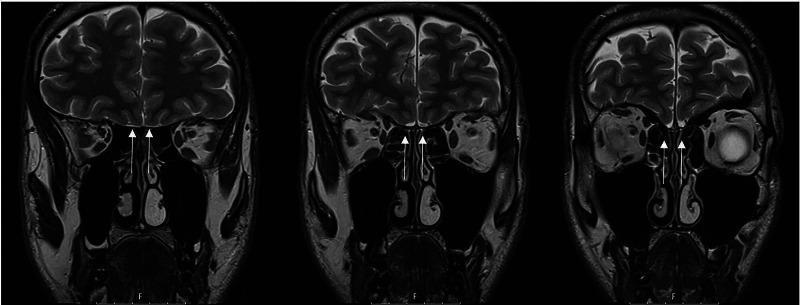
Cerebral and Paranasal Sinuses MRI. Imaging of the paranasal sinuses, olfactory groove and brain confirmed the complete agenesis of both olfactory bulbs (arrows) and did not reveal any other associated nervous or intracranial changes.

We do not have olfactometry at our institution, but an olfactory test was performed with vanilla and freshly ground coffee, without any response. Radiologic investigation started with Computorized Tomography, which was normal, and MRI. Axial and coronal T1W and T2W thin slice olfactory bulb/tract MRI showed an isolated, complete and bilateral agenesis of both olfactory bulbs with no other cerebral and nasal malformations (Figure 1, 2). No parenchymal lesion or abnormal fluid collection was identified.

Hormone studies showed normal levels of growth, adrenocorticotropic, luteinizing, follicle-stimulating and thyroid stimulating hormones, prolactin, testosterone and cortisol. Chest X-ray, complete blood count and blood chemistry were also normal. We were unable to reach any conclusions about fertility because the patient had no children yet and because fertility tests were not carried out.

Given the fact that no curative treatment is available, counselling for daily living precautions especially those related to gas, fire, and rotten food was given to the patient.

## Discussion

Olfactory axons project from nasal epithelium to the primitive telencephalon before OB form. Primordial olfactory bulbs appear at 4.5 weeks (41 days) gestation. However, despite being immature in the term neonate, the OB are already functional and olfactory reflexes are present after 28-32 weeks gestation. So, olfactory maturation remains incomplete at term and has a long course during postnatal development [7].

This case of anosmia is unlikely to have a secondary or acquired origin because there wasn't any event reported by the patient that triggered this complaint and it had been present always, according to the patient. As such, the authors will mainly discuss the differential diagnosis associated with congenital and non-acquired anosmia.

Congenital agenesis of the OB can occur as an isolated defect (isolated congenital anosmia) [1]. On the other hand, it is a constant feature in alobar and semilobar holoprosencephaly, in which there is an incomplete forebrain division [2]. In septo-optic-pituitary dysplasia, a disease characterized by the underdevelopment of the optic nerves, abnormal formation of structures along the midline of the brain, and pituitary hypoplasia, the olfactory bulbs often are hypoplastic [8]. However, in this case we found no abnormalities of the optic tracts and septum. Kallmann’s syndrome is an isolated gonadotropin-releasing hormone deficiency with anosmia and hypogonadism that affects mainly males. Other neurologic findings, which were not present in our patient, such as ataxia, impaired hearing, or mental handicap may also be present [2]. Hatsfield syndrome is a mild form of holoprosencephaly with olfactory bulb agenesis and ectrodactyly (split hand, lobster claw, or cleft hand or foot) but normal physical appearance [2].

Most cases of isolated congenital anosmia occur sporadically in people with no family history of the condition [2]. Only a few sporadic cases have been reported in the literature. Some specific genetic mutations have been associated with this condition [3-5]. Ragancokova et al. (2014) concluded that OB differentiation in mice depended on teashirt zinc finger family member 1 (TSHZ1), which is one of the molecules expressed in the developing olfactory bulbs. The authors highlighted the important role of this gene in human craniofacial development [3].

Despite being rarer than agenesis, supernumerary OB can also be associated with impaired olfaction. Fusion of olfactory bulbs may occur in some cases in which the bulb appears unilaterally absent on gross examination [9,10].

In children and teenagers, the diagnosis is challenging. Smell disorders are rare in childhood and are often underestimated. Obstructive and inflammatory causes like adenoid hypertrophy, hypertrophy of the inferior turbinates, septal deviation, nasal polyps, and juvenile angiofibroma are the most frequent causes of hyposmia at that age. Without any other associated symptom (nasal obstruction, epistaxis, headaches, or rhinorrhea), all of these diagnoses should be excluded, although they are less likely.

Given the loss of warning smell sensation, counselling for daily living precautions should be given. The compensatory mechanism for their inability to detect smells such as the detection of facial expressions by others should be evaluated.

## Conclusions

Kallmann’s Syndrome remains the main differential diagnosis of congenital anosmia. Diagnosis of isolated congenital anosmia with normal sexual characteristics is difficult, especially in adulthood. This case report reminds us of an often forgotten entity and highlights the need to exclude several genetic syndromes that may be associated, albeit in a dissimulated way. Given the loss of warning smell sensation, counselling for daily living precautions should be given to all patients.
